# Influence of Major Environmental Parameters on Patulin Production by *Penicillium expansum* OM1 and Its Growth on Apple Puree Agar Media

**DOI:** 10.3390/toxins18010001

**Published:** 2025-12-19

**Authors:** Haiyi Yu, Sung-Yong Hong, Ji-Yeon Koo, Ae-Son Om

**Affiliations:** Department of Food and Nutrition, College of Human Ecology, Hanyang University, Seoul 04763, Republic of Korea; yuhaiyi@naver.com (H.Y.); lunohong@hanyang.ac.kr (S.-Y.H.); goo0624@naver.com (J.-Y.K.)

**Keywords:** apple puree agar media, patulin, temperature, pH, relative humidity, *Penicillium expansum* OM1

## Abstract

Patulin is a mycotoxin produced mainly by *Penicillium expansum* on apples. *P. expansum* is a fruit pathogen that can cause apple soft rot. However, much is unknown about the characteristics of *P. expansum* and influence of major environmental parameters on its patulin production and growth on apple puree agar media (APAM). In this study, we evaluated the influence of pH, temperature, and relative humidity (RH) on patulin production by *P. expansum* OM1 and its growth (colony diameter and mycelial dry weight) on APAM after isolation and identification of the patulin-producing fungal strain from an apple. The fungal isolate produced the largest quantity of patulin on APAM under 15 °C, pH 4.0, and RH 98%, while it had the highest growth rates on the same media under 25 °C, pH 4.0–6.0, and RH 98%. Our data demonstrated that three important physicochemical factors (pH, temperature, and RH) substantially influenced the patulin production by the fungal species and its growth on APAM. Moreover, our results revealed that patulin was not detected on APAM at 5 °C after 7 days of incubation and that a trace amount of patulin was produced by the fungal strain along with its slow growth on the same media at 5 °C after 14 days. It suggests that patulin contamination by *P. expansum* on apples could be controlled during postharvest storage below 5 °C. These findings could provide fundamental knowledge for development of efficient strategies to prevent the occurrence of apples contaminated with patulin produced by *P. expansum* on them during postharvest storage.

## 1. Introduction

Patulin is a biologically active toxic metabolite produced by certain species of *Penicillium* and *Aspergillus* mainly on fruits, vegetables, and their derived products, including apples, pears, and peaches [1,2]. Patulin contamination on apple fruits is usually associated with apple soft rot caused by *Penicillium expansum* (*P. expansum*) [1,3]. The patulin contamination on apples and apple products poses a high health risk to consumers since it can cause serious acute and chronic toxic effects in humans [4]. Assessment of the human health risk suggests that exposure to patulin can cause acute symptoms such as hyperemia, inflammation, edema, ulceration, intestinal hemorrhage, and gastrointestinal and kidney damage [1,5,6]. Patulin has also been associated with immunotoxicity, neurotoxicity, genotoxicity, teratogenicity, and carcinogenicity as the chronic toxic effects [5,6,7]. The patulin toxicity is believed to arise from adduct formation with thiol-containing biological components, including glutathione and cysteine-containing proteins, and induction of intra- and intermolecular cross-links in proteins by reacting with the thiol group of cysteine as well as oxidative stress generation [2,5,8]. As a result, it can produce disruption of protein synthesis, organelle dysfunction, and inhibition of translation and transcription [2,5]. Hence, a permissible regulatory limit (50 µg/L) of PAT in apples and apple-based products has been established by the Codex Alimentarius Commission (CAC), European Commission (EC), and the US Food and Drug Administration (FDA) [9,10,11].

It has been documented that both patulin production and fungal growth can be affected by some physicochemical factors such as pH, relative humidity (RH), and temperature [12,13,14,15,16]. In addition, many researchers have reported physiological characteristics of *P. expansum* for patulin production on several different types of media such as Czapek glucose agar medium (CGAM), potato dextrose agar (PDA), or malt extract agar (MEA) containing sucrose or glycerol [12,13,16]. However, much is unknown about the key environmental factors for patulin production on apple-based media by *P. expansum*. Hence, in our study, we isolated patulin-accumulating *P. expansum* OM1 from an apple and evaluated the culture conditions under which its patulin production and growth on apple-based media are inhibited by incubation at different temperature, pH, and RH. Our data demonstrated that the largest quantity of patulin was produced by *P. expansum* OM1 on apple puree agar media (APAM) at 15 °C, pH 4.0, and RH 98%, whereas the highest growth rate of the strain on the same media was at 25 °C, pH 4.0–6.0, and RH 98%. In addition, our results showed a very small quantity of patulin was produced by the strain on APAM at 5 °C on the 14th day. It indicates that the occurrence of patulin contaminated-apples due to *P. expansum* OM1 could be prevented when they are stored below 5 °C. The present study could help control the growth of *P. expansum* on apples and their patulin contamination by the fungal species.

## 2. Results and Discussion

### 2.1. Screening for Patulin-Producing Fungi and Their Identification

**Figure 1:**
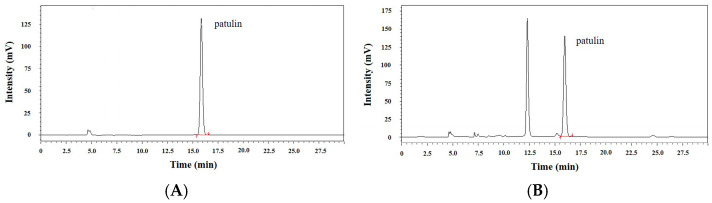
Chromatograms of patulin analyzed by HPLC. Chromatograms of (**A**) patulin standard (2.0 μg/mL) and (**B**) extracts from culture of isolate 36 for patulin analyses. Patulin was eluted at 15.9 min.

In total, 104 fungal strains were isolated from the rotten surface of four apples with blue mold symptoms. The fungal isolates were analyzed for patulin production using high-performance liquid chromatography (HPLC). Thirty-nine of them were able to produce patulin (0.02–2.09 μg/mL; above a limit of quantification (LOQ; 0.018 μg/mL) from all 39 isolates). Then, 7 fungal isolates, which showed high patulin production, from the 39 fungi were selected and identified genetically through sequencing of ITS1-5.8S rDNA-ITS2 region on fungal rDNA. Polymerase chain reaction (PCR) successfully amplified the ITS region on genomic DNA of all seven fungal strains, and we compared the nucleotide sequences in the PCR products from the fungal isolates with those of reference strains, which were fetched from the GenBank database at the National Center for Biotechnology Information (NCBI) website, using nucleotide Basic Local Alignment Search Tool (BLASTn, https://blast.ncbi.nlm.nih.gov/, accessed on March 2022). The assessment using BLASTn revealed that there is a high DNA sequence alignment (96–100%) between the two sequences of the fungal strains and the reference strains (Table 1). All 7 isolates were identified as *P. expansum*. Of seven fungal isolates, one isolate (36) produced the largest quantity of patulin (2.09 μg/mL) (Table 1, Figure 1A,B). We then selected the fungal isolate 36, and designated it as *P. expansum* OM1. Figure S2 shows the morphological characteristics of *P. expansum* OM1 on four types of agar plates (APAM, PDA, MEA, and YES).

**Figure 2:**
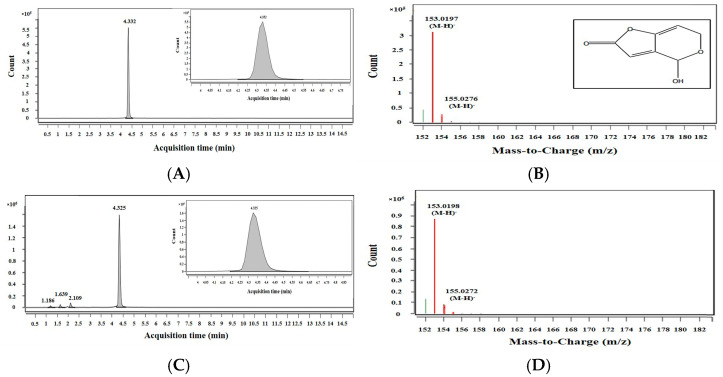
Extracted ion chromatograms (EIC) and mass (MS) spectra of patulin. (**A**) EIC and (**B**) MS spectrum for patulin in a patulin standard (2 μg/mL), and (**C**) EIC and (**D**) MS spectrum for patulin in culture extracts of *P. expansum* OM1. (Inset) (A) Magnification of the patulin peak from the patulin standard, (**B**) patulin structure, and (**C**) magnification of the patulin peak from fungal culture extracts from *P. expansum* OM1.

The patulin production by *P. expansum* OM1 was confirmed using liquid chromatography/Quadrupole Time-of-Flight (LC/Q-TOF) analysis. The patulin standard and that in the culture extracts of the strain were eluted at 4.332 min and 4.325 min, respectively (Figure 2A,C). In addition, the mass-to-charge (*m*/*z*) ratio of the major [M-H]^−^ ion related to the peak in the patulin standard was 153.0197, and the *m*/*z* ratio of the main [M-H]^−^ ion related to the peak in the culture extracts of the strain was 153.0198 (Figure 2B,D). They were almost identical, and the mass (MS) profiles were a close match. These data verified that *P. expansum* OM1 produced patulin.

### 2.2. Influence of Temperature on the Amounts of Patulin Produced by P. expansum OM1 and Growth Rates of the Strain

**Figure 3:**
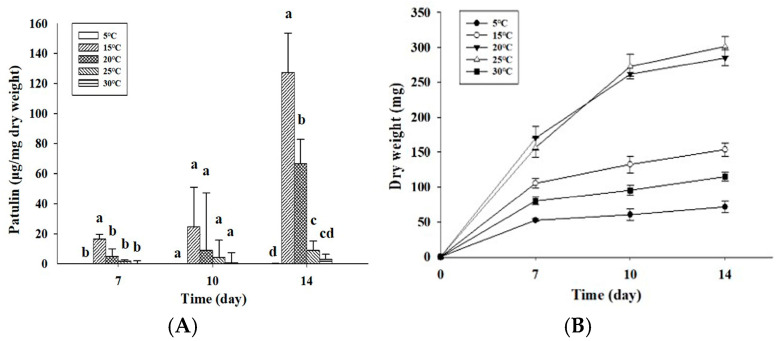
Patulin levels produced by *P. expansum* OM1 and its growth rates on APAM under 5 temperature conditions (5, 15, 20, 25, and 30 °C) for 14 days. (**A**) Patulin levels and (**B**) mycelial dry weight. The patulin levels and mycelial dry weight were measured in triplicate. Data are represented as the mean ± standard deviation. Different letters in the same group indicate statistically significant differences (*p* < 0.05).

Temperature plays an important role in mycotoxin levels produced by fungi in relation to their growth [12]. In particular, it is known that *P. expansum* has a temperature range for patulin production of 0–24 °C [13]. Also, there are some contradictory results in the optimal conditions for patulin levels produced by different *P. expansum* isolates and for their growth on different media [12,17,18]. Therefore, in order to evaluate the influence of temperature on the amounts of patulin produced by *P. expansum* OM1, the fungal isolate was cultured on APAM under five temperature conditions (5, 15, 20, 25, and 30 °C). The amounts of patulin produced by the fungal isolate were temperature dependent, and the levels of patulin showed a typical bell shape over five different temperature conditions (Figure 3A). The fungus produced the largest quantity of patulin at 15 °C (127.28 μg/mg dry weight) on the 14th day of incubation, which was followed by 20 °C (66.48 μg/mg dry weight), 25 °C (8.87 μg/mg dry weight), and 30 °C (3.13 μg/mg dry weight). In particular, the fungus produced a very small amount of patulin at 5 °C (0.10 μg/mg dry weight) on the 14th day, whereas patulin was not detected at 5 °C after 7 days of incubation (Figure 3A). It indicates that patulin contamination of apples can occur at 5 °C after a long period of refrigeration. Our data are in good agreement with those from previous studies [12,13,19]. A previous study from Lebanon reported that *P. expansum* NRRL 35695 accumulated the largest quantity of patulin on CGAM at 16 °C among three different temperature conditions (8, 16, and 25 °C) [12]. Another study described that the largest quantity of patulin was produced at 16 °C by 2 *P. expansum* strains on MEA supplemented with sucrose or glycerol [13]. Paster et al. (1995) also showed that the optimal temperature for patulin production of *P. expansum* strains on apples was 17 °C among three different storage temperatures (6, 17, and 25 °C) [19]. However, in their study, the second highest level of patulin production was strain dependent, and two strains produced the second highest level at 6 °C, while one strain produced the second highest level at 25 °C, which is similar to our results. In contrast, some previous studies documented slightly different results from our study [17,18]. Baert et al. (2007) reported that strain-dependent variability in patulin production in their study, in which the largest quantity of patulin on APAM were produced by certain *P. expansum* strains at 4 °C, while the highest levels on the same media were produced by the other strains at 10 °C among three different temperature conditions (4, 10, and 20 °C) [17]. Another study showed that the largest quantity of patulin in PDB was produced by one *P. expansum* strain at 25 °C after 14 days [18].

Next, the growth rates of *P. expansum* OM1 on APAM plates were analyzed under five different temperature conditions (5, 15, 20, 25, and 30 °C). The growth rate of the fungal strain on APAM was highest at 25 °C among the five different temperature conditions on the 14th day of incubation (dry weight, 301.42 mg) (Figure 3B). The growth rate at 20 °C was similar to that at 25 °C, but it decreased slightly relative to that at 25 °C. Also, the fungal growth rate at 15 °C was much slower than that at 25 and 20 °C, and it was followed by the rate at 30 °C. These results were similar in the growth patterns to the data based on colony diameter. The fungal growth, which was based on colony diameter, was highest at 25 °C after 12 days (colony diameter, 80.67 mm) (Figure S3). The growth showed a rapid increase at 25 and 20 °C after 2 days of incubation, whereas it showed a steady increase at 15 °C over 12 days of incubation. Moreover, the fungal growth rate was remarkably retarded at 30 and 5 °C for 12 days, and it showed a slow increase at 30 °C after a lag phase for 2 days, whereas it showed a slow increasing tendency at 5 °C after a lag period for 6 days (Figure S3). These data support that cold storage of apples at 5 °C just slows down the growth of *P. expansum* without preventing it completely [12,20]. Our results suggest that the optimal temperature for growth of *P. expansum* OM1 on APAM is 25 °C, which aligns with the results from previous research [12,13,17,21]. One previous study documented that *P. expansum* typically exhibits the optimal temperature for its growth in the range of 20 to 25 °C [21]. Another study also reported that a *P. expansum* strain grew best on APAM at 25 °C among four different temperature conditions (16, 20, 25, and 30 °C) [17]. In addition, in Tannous and co-workers’ study, *P. expansum* NRRL 35695 on CGAM had the optimum growth temperature at 25 °C although it was isolated from grapes and was cultured on a different medium from that in our study [12]. A study from the Netherlands also reported that 2 *P. expansum* strains had the highest growth at 24 °C among three different temperature conditions (16, 24, and 31 °C) [13]. However, growth data from a previous study showed slightly different results from our study [18]. In their study, *P. expansum* isolated from a pear had the highest growth in potato dextrose broth (PDB) at 30 °C after 14 days of incubation. In contrast, our data indicate that the relatively high temperature (30 °C) inhibits fungal growth as well as patulin production on APAM when taken together with the results on the patulin levels described above. Overall, *P. expansum* OM1 produced the largest quantity of patulin at 15 °C on APAM, whereas it had the optimum growth rate at 25 °C on APAM, indicating that the optimum temperature for growth of *P. expansum* OM1 was higher than that for its patulin production. This is in accordance with the results from previous research. Several previous studies have reported that the optimum condition for mycotoxin production was different from the most favored condition for fungal growth [12,13,17]. Moreover, in some previous studies the preferred temperature conditions for growth of *P. expansum* were higher than those for patulin production of the strains [13,17,18]. Based on the patulin biosynthetic pathway, we assume that increased amounts of acetyl CoA, an early precursor for patulin biosynthesis, enter TCA cycle for fungal growth instead of entry into the patulin biosynthetic pathway when incubation temperature increases to above 15 °C (15–30 °C), resulting in decreased amounts of patulin production.

### 2.3. Influence of pH on the Amounts of Patulin Produced by P. expansum OM1 and Growth Rates of the Strain

**Figure 4:**
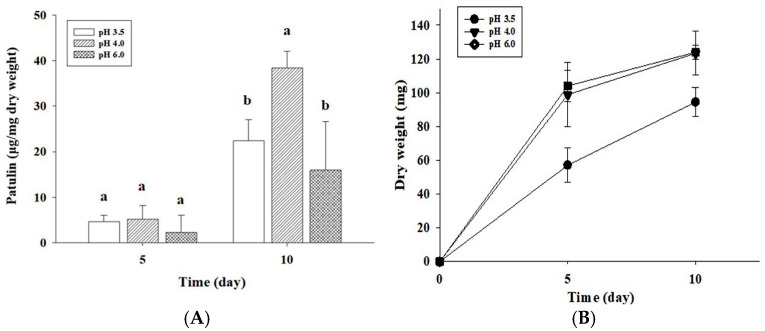
Patulin levels produced by *P. expansum* OM1 and its growth rates on APAM under 3 different pH conditions (pH 3.5, 4.0, and 6.0) at 15 °C for 10 days. (**A**) Patulin levels and (**B**) mycelial dry weight. The patulin levels and mycelial dry weight were measured in triplicate. Data are represented as the mean ± standard deviation. Different letters in the same group indicate statistically significant differences (*p* < 0.05).

The environmental pH can affect not only fungal growth but also mycotoxin production by changes in metabolic enzyme activities and mycotoxin biosynthetic gene expression through proton gradients across cell membranes [16,22,23]. Thus, first we analyzed the patulin levels produced by *P. expansum* OM1 on APAM plates at three different pH levels (pH 3.5, 4.0, and 6.0) at 15 °C. The fungus produced the largest quantity of patulin at pH 4.0 after 10 days of incubation (38.45 μg/mg dry weight), which was followed by the patulin levels at pH 3.5 (22.45 μg/mg dry weight) and pH 6.0 (15.98 μg/mg dry weight) (Figure 4A). However, any statistically significant difference was not observed between patulin levels at pH 3.5 and 6.0. Our data are in good agreement with those from previous studies [12,16,24]. One previous study described that the largest quantity of patulin was produced at pH 3.2–3.8 by a *P. expansum* strain in apple juice [24]. Another study showed that *P. expansum* NRRL 35695 on CGAM produced the largest quantity of patulin at pH 4.0 among three different pH (pH 2.5, 4.0, and 7.0) [12]. Jimdjio et al. (2021) also reported that the largest quantity of patulin was produced at pH 5.0 by *P. expansum* T01 on PDA or an apple among four pH (pH 2.5, 5.0, 7.0, and 8.5) [16]. However, the results in our study slightly differ from one study, in which the authors described that the largest quantity of patulin was produced by *P. expansum* in apple juice at pH 3.5 among four pH (pH 3.0, 3.5, 4.0, and 5.0) [25]. One of the possible reasons for this may be the use of different strain or medium.

On the other hand, two previous studies reported that patulin is remarkably stable at pH 3.5–5.5 [12,26]. In addition, it is known that genes that encode for PacC (a pH-dependent global activator for secondary metabolism) and PatL (a pathway-specific transcription factor for patulin production) are up-regulated for patulin production by *P. expansum* under acidic conditions [16,27,28]. Thus, it is likely that larger quantity of patulin is produced by *P. expansum* under acidic pH than alkaline pH.

In addition, the growth rates of *P. expansum* OM1 on APAM plates were analyzed at three different pH (pH 3.5, 4.0, and 6.0) at 15 °C, at which the fungus exhibited the largest quantity of patulin production as described above. *P. expansum* OM1 displayed a similar growth rate at pH 6.0 to that at pH 4.0 over 10 days (dry weight, 124.00 mg at pH 6.0; dry weight, 123.37 mg at pH 6.0) (Figure 4B). However, the fungal growth rate at pH 3.5 (dry weight, 94.43 mg) was much slower than that at pH 4.0 or pH 6.0. These results were slightly different from the growth patterns based on colony diameter. The fungal growth rates, which were based on colony diameter, did not show statistically significant difference among those under three different pH conditions (Figure S4). One of the possible reasons for this may be that this fungal strain grows more sparsely at pH 3.5 than pH 4.0 and 6.0. Also, fungal growth rates at 25 °C showed a similar tendency to those at 15 °C. The growth rates based on dry weight and colony diameter were highest at pH 4.0 and 6.0 among three pH conditions (Figure S5A,B). Our data suggest that the optimal growth pH of *P. expansum* OM1 on APAM is pH 4.0–6.0. These results are in accordance with the data from previous studies [12,16,29]. Li et al. (2020) reported that pH 4.0–5.0 is optimum for growth of *P. expansum* [29]. One previous study from China documented that the highest growth rates (mycelial dry weight and colony diameter) of *P. expansum* T01 on PDB or PDA were at pH 5.0 among four different pH (pH 2.5, 5.0, 7.0, and 8.5) [16]. Another study showed that *P. expansum* NRRL 35695 on CGAM had the highest growth rate (colony diameter and dry weight) at pH 4 among three different pH conditions (2.5, 4.0, and 7.0) [12]. In both previous studies, the second highest growth rate was at pH 7.0, which is similar to the data from our study.

Considering the results described above, our results indicate that *P. expansum* OM1 grows well on APAM under weak acidic conditions (pH 4.0–6.0) including pH of natural apples (pH 4.4) and that the largest quantity of patulin was accumulated by the fungal species on the same media at pH 4.0. Overall, *P. expansum* OM1 showed the largest quantity of patulin production at pH 4.0 and 15 °C on APAM, whereas the species showed the optimum growth rate at pH 4.0–6.0 and 25 °C on the same media.

### 2.4. Influence of RH on the Amounts of Patulin Produced by P. expansum OM1 and Growth Rates of the Strain

**Figure 5:**
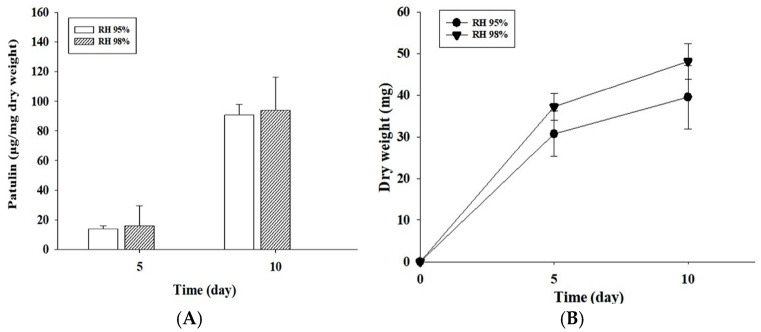
Patulin levels produced by *P. expansum* OM1 and its growth rates on APAM (pH 4.0) at 2 RH (95 and 98%) and 15 °C for 10 days. (**A**) Patulin levels and (**B**) mycelial dry weight. The patulin levels and mycelial dry weight were measured in triplicate. Data are represented as the mean ± standard deviation.

RH also influences fungal survival, growth rates, and its mycotoxin production. Hence, we evaluated the patulin levels produced by *P. expansum* OM1 and growth rates of the fungus on APAM agar plates (pH 4.0) at 2 RH (95 and 98%) at 15 °C. The fungus produced the slightly higher amount of patulin under RH 98% condition (93.89 μg/mg dry weight) than under RH 95% condition (90.77 μg/mg dry weight) on the 10th day (Figure 5A). However, no statistically significant difference was observed between patulin levels under RH 95 and 98% (*p* > 0.05). Thus, our results imply that *P. expansum* OM1 grows slowly and produced decreased amounts of patulin under the lower RH (95%) than higher RH (98%) although there was no statistical difference between the patulin levels under the two conditions. Our data slightly differ from those from other previous studies [12,13,30]. A previous study documented that *P. expansum* NRRL 35695 produced larger quantity of patulin on CGAM under water activity (a_w_) 0.99 than under a_w_ 0.90 or 0.95 after 14 days at 25 °C and that only trace amounts of patulin were detected under a_w_ 0.90 or 0.95 [12]. Also, Lindroth et al. (1978) documented that the minimum a_w_ for production of patulin from one *P. expansum* strain on blueberries or strawberries was 0.94, at which very small amounts of patulin were produced [30]. In contrast, Northolt and collaborators showed that the minimum a_w_ by two *P. expansum* strains on MEA supplemented with sucrose or glycerol was 0.99 [13]. It suggests that the limiting a_w_ for production of patulin from *P. expansum* is strain- and media-dependent. Additionally, the fungal strain showed higher growth rates under RH 98% condition (48.10 mg) than under RH 95% condition (39.50 mg) after 10 days (Figure 5B). The growth based on fungal colony diameter also exhibited a similar pattern to that based on dry weight (Figure S6). Overall, when taken together with the above results, the largest quantity of patulin was produced by *P. expansum* OM1 on APAM at pH 4.0, 15 °C, and RH 98%, while the strain on the same media showed the highest growth rate at pH 4.0–6.0, 25 °C, and RH 98%. Our results also demonstrated that the three major environmental parameters including pH, temperature, and RH remarkably influenced the patulin levels produced by *P. expansum* OM1 and the growth rate of the fungal strain.

## 3. Conclusions

This study aimed to investigate the influence of temperature, pH, and RH on the patulin levels produced by *P. expansum* OM1 and the growth rates of the strain on APAM, apple-based media, after isolation of the patulin-producing *P. expansum* from an apple. Our results demonstrated that the three key environmental parameters greatly influenced the patulin levels produced by the fungus and its growth on APAM. In addition, it revealed that the largest quantity of patulin produced by *P. expansum* OM1 on APAM was observed at 15 °C, pH 4.0, and RH 98%, whereas the highest growth rate of the strain on the same media was at 25 °C, pH 4.0–6.0, and RH 98%. In particular, our data showed that a trace amount of patulin was produced by the strain on APAM until the 14th day of incubation at 5 °C. These findings suggest that patulin production by *P. expansum* OM1 on apples could be controlled during its postharvest storage below 5 °C. The present study could help prevent the patulin contamination of apples by *P. expansum* on them during storage.

## 4. Materials and Methods

### 4.1. Chemicals

Disodium ethylenediaminetetraacetic acid (Na_2_EDTA), ethanol, proteinase K, Tween 80, acetic acid (≥98.0% purity), and patulin standard (≥98.0% purity) were obtained from Sigma-Aldrich Co. (St. Louis, MO, USA), and ethyl acetate was from Daejung chemicals and metals Co. (Seoul, Republic of Korea). Tris base, sodium dodecyl sulfate (SDS), and 2-mercaptoethanol were purchased from Bio rad (Hercules, CA, USA), and sodium carbonate (Na_2_CO_3_) was from Samchun chemical Co., Ltd. (Seoul, Republic of Korea). Anhydrous sodium sulfate (Na_2_SO_4_), sodium acetate, K_2_SO_4_, and KNO_3_ were obtained from Junsei chemical Co., Ltd. (Tokyo, Japan), and phenol/chloroform/isoamyl alcohol (v:v:v = 25:24:1) was from Biochemicals Inc. (Gyeonggi, Republic of Korea). Acetonitrile (ACN, ≥98.0% purity) was purchased from J. T. Baker chemical Co., Ltd. (Phillipsburg, NJ, USA).

### 4.2. Isolation of Fungi from Apples

Rotten apples (cultivar Fuji), which were stored at fruits and vegetables shops in the local market (Seoul, Republic of Korea), were collected in the winter of 2021, and used to isolate patulin-producing fungi. After several blue mold spots on the apples were touched with sterilized cotton swabs using a swab method, the swab tips were individually dipped in sterilized saline solutions (5 mL). After being mixed, each cell suspension was diluted and inoculated onto a PDA (MB Cell, Seoul, Republic of Korea) plate supplemented with chloramphenicol and tetracycline (5 mg of chloramphenicol and 5 mg of tetracycline per 1 L PDA). Then, the agar plates were incubated at 30 °C until colonies were formed. Each fungal colony was subcultured on a fresh PDA plate and further incubated at 30 °C for 4 days. Isolation of pure strains was performed by inoculation of spores onto new PDA plates after the spore suspensions were prepared with 0.01% Tween 80 and diluted with sterile distilled water (DW).

### 4.3. Genetic Identification of Fungal Strains Isolated from Apples

Fungal strains, which were isolated from apples, were genetically identified using DNA sequencing of the internal transcribed spacer 1 (ITS1)-5.8S rDNA-ITS2 region on fungal rDNA [31].

For genomic DNA isolation, after fungal spores were prepared from fungal strains grown on PDA plates, 10^7^ spores were inoculated into 100 mL of PDB (MB Cell, Seoul, Republic of Korea) in a 250 mL flask and incubated at 30 °C for 4 days under agitation at 150 rpm. Fungal mycelia were then filtered through miracloth (Sigma-Aldrich Co., St. Louis, MO, USA) on a Buchner funnel (Daihan Scientific, Wonju, Gangwon, Republic of Korea), and genomic DNA isolation from the fungal mycelia was performed by a method of Steven B Lee and John W. Taylor using phenol/chloroform/isoamyl alcohol with some modifications [32]. The ITS region of the isolated genomic DNA was then amplified by PCR using a pair of specific primers (ITS1 and 4) for identification of fungal species. The primer sequences are as follows: ITS1 (5′-TCCGTAGGTGAACCTGCGG-3′, forward) and ITS4 (5′-TCCTCCGCTTATTGATATGC-3′, reverse). PCR was conducted at 95 °C for 5 min, followed by 35 cycles of 95 °C for 1 min (denaturation), 55 °C for 1 min (annealing), and 72 °C for 2 min (extension), and 72 °C for 10 min (final extension). The PCR products were run on 1.2% (*w*/*v*) agarose gels by electrophoresis, and purified using AccuPep PCR/Gel Purification Kit (Bioneer, Daejeon, Republic of Korea). The purified PCR products were sequenced by Biofact Co. (Daejeon, Republic of Korea). Then, the fungal isolates were identified by the local similarity between DNA sequences of the PCR products and those of fungal strains, which were retrieved from GenBank at the NCBI) website.

### 4.4. Fungal Culture Media and Incubation Conditions

For isolation of patulin-producing fungal strains, spores (10^6^) were inoculated into PDB (5 mL) for 5-day incubation at 30 °C under agitation conditions at 120 rpm after spore preparation with 0.01% Tween 80.

For fungal culture on APAM, 2 × 10^4^ spores were inoculated onto each APAM agar plate, which was overlaid with a sterile cellophane membrane (Bio-rad, Hercules, CA, USA), after preparation of APAM agar plates including 40% apple puree by mixing homogenized apples (cultivar Fuji) with DW (homogenized apples: DW = 1:1.5 [*w*/*w*]) and adding agar into it. Then, for fungal culture at different temperature, the APAM plates were incubated at 5 different temperatures (5, 15, 20, 25, and 30 °C) for 14 days.

For culture at 3 different pH (pH 3.5, 4.0, and 6.0), pH of APAM agar plates (pH 4.4, unadjusted pH) was adjusted by adding HCl or NaOH. After 2 × 10^4^ spores were inoculated, the APAM agar plates were incubated at 25 or 15 °C for 10 days.

For culture at 2 different relative humidity (RH), APAM agar plates (pH 4.0) were placed in sealed plastic bins (200 mm × 150 mm × 80 mm; Daiso, Seoul, South Korea; 6 APAM agar plates in one plastic bin), and the plastic bins were adjusted to 95 or 98% RH with saturated salt solutions (KNO_3_ for 95% RH, K_2_SO_4_ for 98% RH at 15 °C) [33,34,35]. Then, the bins were incubated at 15 °C for 10 days.

For observation of fungal morphology, spores (2 × 10^4^) were inoculated onto APAM, PDA, MEA (2% malt extract, 0.1% peptone, 2% dextrose), or yeast extract sucrose (YES; 2% yeast extract, 0.1% MgSO_4_·7H_2_O, 15% sucrose) agar plates for 5-day incubation at 25 °C. Then, fungal morphological characteristics were observed under a microscope (Olympus IX 71, Olympus Co., Ltd., Tokyo, Japan) after preparation of slides using lactic acid.

### 4.5. Assessment of Fungal Growth Rates

The growth rates of the fungal strain were determined by measuring the colony diameter and mycelial dry weight grown on APAM agar plates. Dry weight of mycelia was assessed after the mycelia cultured on APAM agar plates, which were overlaid with cellophane membranes, were fully dried at 80 °C. Radial growth rates were evaluated by measurement of the two diameters of the colony along the perpendicular direction [12].

### 4.6. Preparation of Patulin Standard Solutions and Patulin Extraction from Fungal Culture

A patulin stock solution (200 μg/mL) was prepared by dissolving patulin (5 mg) in ethyl acetate (25 mL) and stored at −20 °C until it was needed. Five levels of patulin standard solutions with concentrations of 0.1, 0.2, 0.5, 1.0, and 2.0 μg/mL were freshly made by diluting the patulin stock solution with ethyl acetate. After each patulin standard solution (1 mL) was completely dried under N_2_ at 60 °C, the dried residue was dissolved in acidified DW (1 mL; pH 4.0, adjusted with acetic acid).

Patulin extraction from fungal culture was carried out following the AOAC method 995.10 with minor modifications [36] as described previously [37]. Briefly, after each fungal culture was extracted twice with ethyl acetate and washed with 1.5% sodium carbonate solution, it was dried over anhydrous sodium sulfate and evaporated to complete dryness under nitrogen at 60 °C. The dried residue was then dissolved in 1 mL of acidified DW (pH 4.0), and a 0.2 μm polyvinylidone fluoride (PVDF) syringe filter (Hyundai Micro Co., Ltd., Seoul, Republic of Korea) was used to filter the dissolved solution before injection into an HPLC system.

### 4.7. HPLC Analysis

An HPLC system (LC-20AD, Shimadzu; Tokyo, Japan), which was equipped with UV detector (UVD, SPD-20A, Shimadzu; Tokyo, Japan), was used to detect and quantify patulin at 276 nm. The analytes were separated on a ZORBAX Eclips plus C18 column (5 μm particle size, 4.6 mm x 250 mm, Agilent; Santa Clara, CA, USA). The mobile phase consisted of 10% ACN (ACN: DW = 10:90, *v*/*v*) with a constant flow rate of 0.5 mL/min, giving a total run time of 30 min. The injection volume of the samples was 100 μL, and the column temperature was set at 25 °C.

The linear relationship between five levels of patulin in the HPLC-UVD analysis was evaluated by construction of a calibration curve using the patulin standard solutions with concentrations of 0.1, 0.2, 0.5, 1, and 2 μg/mL. The patulin standard curve was created by plotting the patulin levels (*x* axis) against the peak areas (*y* axis) in the analytical method. The linearity of the calibration curve was analyzed using linear regression and determined by a coefficient of determination (r^2^). The r^2^ value of the standard curve using patulin standard solutions was 0.999 (Figure S1).

To assess the sensitivity of the HPLC-UVD analysis for patulin, LOQ and a limit of detection (LOD) were used. LOQ and LOD were calculated using the slope (S) of the standard curve and the standard deviation (SD) of the response, which were gathered through linear regression analyses, as follows:

$$LOQ=\frac{10\times SD}{S}$$

$$LOD=\frac{3.3\times SD}{S}$$

The LOQ for patulin was 0.018 μg/mL, and the LOD for the toxin was 0.006 μg/mL.

### 4.8. LC/Q-TOF Analysis

The identity of patulin was confirmed by LC/Q-TOF. The LC/Q-TOF analysis was carried out using an Agilent 1290 Infinity UHPLC system (Santa Clara, CA, USA), which was connected to an Agilent 6545XT LC/6550 iFunnel Q-TOF LC-MS that incorporates a dual-spray Agilent Jet Stream electrospray ionization (ESI) source. Compounds in samples were separated on Agilent ZORBAX Eclipse Plus C18 column (1.8 μm particle size, 2.1 mm × 100 mm) with a mobile phase at a flow rate of 0.25 mL/min. One mobile phase (A solution) was composed of 100% pure DW, whereas another mobile phase (B solution) was composed of 100% ACN. The details of the applied gradient elution program are as follows: after B solution was held on 5% for 2 min, it linearly increased from 5% to 50% for 4 min. Then, it continuously increased from 50% to 90% for 0.5 min and was maintained at 90% for 2 min. Subsequently, the 90% of B solution was rapidly decreased from 90% to 5% for 1 min. The injection volume of samples was 10 μL. The column oven temperature was maintained at 40 °C.

The MS spectrometer was operated in the negative ESI mode, and the peak spectrum was obtained by the Find by Formula data-mining algorithm. The main MS parameters were optimized and set as follows: mass range, 50 to 500 amu; scan rate, 1 spectra/sec; capillary voltage, 3500 V; drying gas temperature, 325 °C; drying gas flow rate, 6 L/min; sheath gas temperature, 350 °C; sheath gas flow rate, 11 L/min; nebulizer gas pressure, 45 psi; skimmer, 65 V; and octupole radiofrequency voltage, 750 V. Agilent MassHunter Data Acquisition Software, rev. 10.0 (Santa Clara, CA, USA) was employed for data processing.

### 4.9. Statistical Analyses

Statistical analyses for comparison between more than 3 samples were performed by a one-way analysis of variance (ANOVA), which was followed by Duncan’s test as a post hoc analysis. Data were represented as the mean ± SD using SPSS 26.0 (SPSS Inc., Chicago, IL, USA). Also, statistical analyses for comparison between 2 samples were performed by Student’s *t*-test using SigmaStat scientific statistical software (version 1.0, Jandel corporation, San Rafael, CA, USA). A *p* value < 0.05 was considered statistically different.

## Figures and Tables

**Table 1:** Amounts of patulin produced by 7 fungal strains, DNA sequence similarity in ITS region, and scientific names of the fungal isolates identified by BLASTn.

Sample ID	Isolate or Strain no. from NCBI	Scientific Name (BLASTn Accession No.) ^1^	Sequence Similarity (%)	Patulin (μg/mL) ^2^
32	Isolate SE1	*Penicillium expansum* (MK201595.1)	99.79	1.71 ± 0.14 ^a^
36	Isolate PF-022	*Penicillium expansum* (MN752156)	97.22	2.09 ± 0.03 ^b^
41	Strain Pe1614M	*Penicillium expansum* (KP670440)	99.62	0.51 ± 0.07 ^c^
44	Isolate 2010F13	*Penicillium expansum* (MT558929.1)	98.62	1.41 ± 0.06 ^d^
47	Isolate SE1	*Penicillium expansum* (MK201595.1)	98.45	1.18 ± 0.11 ^e^
53	Strain HBPe5	*Penicillium expansum* (MH152321.1)	96.60	0.67 ± 0.04 ^f^
56	Strain AL1	*Penicillium expansum* (OR052528.1)	95.74	0.42 ± 0.05 ^c^

^1^ BLASTn was performed using ITS1-5.8S rDNA-ITS2 sequences. BLASTn represents basic local alignment search tool for nucleotide, whereas ITS indicates internal transcribed spacer. ^2^ Data are represented as the mean ± standard deviation. Different letters indicate statistically significant differences (*p* < 0.05).

## Data Availability

The original contributions presented in this study are included in the article/Supplementary Material. Further inquiries can be directed to the corresponding author.

## Supplementary Materials

The following supporting information can be downloaded at: https://www.mdpi.com/article/10.3390/toxins18010001/s1, Figure S1: Calibration curve of patulin standard solutions for patulin quantification by HPLC. Five levels of patulin standard solutions were prepared in the range of 0.1, 0.2, 0.5, 1.0, and 2.0 μg/mL. Each solution was injected into HPLC-UVD in triplicate; Figure S2: Morphological characteristics of *P. expansum* OM1 on 4 different culture media. (A) Colonies on APAM agar plates, (B) colonies on PDA plates, (C) colonies on MEA plates, (D) colonies on YES agar plates, (E) conidiospores and conidiophore on APAM agar plates (400×), (F) conidiospores and conidiophores on PDA plates (400×), and (G) conidiophores on MEA plates (400×). Left photographs show top views, while right photographs show bottom views in (A–D). Scale bar, 100 μm; Figure S3: Colony diameters of *P. expansum* OM1 on APAM agar plates under 5 different temperature conditions (5, 15, 20, 25, and 30 °C) for 12 days. The radial colony diameter was measured in triplicate. Data are represented as the mean ± standard deviation; Figure S4: Colony diameters of *P. expansum* OM1 on APAM agar plates under 3 different pH conditions (pH 3.5, 4.0, and 6.0) and 15 °C for 10 days. The radial colony diameter was measured in triplicate. Data are represented as the mean ± standard deviation; Figure S5: Growth rates of *P. expansum* OM1 on APAM agar plates under 3 different pH conditions (pH 3.5, 4.0, and 6.0) and 25 °C for 10 days. (A) Dry weight and (B) colony diameter. The dry weight and colony diameter were measured in triplicate. Data are represented as the mean ± standard deviation. Figure S6: Colony diameters of *P. expansum* OM1 on APAM agar plates (pH 4.0) at 2 different RH (95 and 98%) and 15 °C for 10 days. The radial colony diameter was measured in triplicate. Data are represented as the mean ± standard deviation.

## Abbreviations

The following abbreviations are used in this manuscript:

APAM	Apple puree agar media
ITS	Internal transcribed spacer
RH	Relative humidity
