# Supplementary material for: Influence of Major Environmental Parameters on Patulin Production by Penicillium expansum OM1 and Its Growth on Apple Puree Agar Media
Source: Toxins (Basel). 2025 Dec 19;18(1):1. doi: 10.3390/toxins18010001 (PMC12846605; doi:10.3390/toxins18010001)
Supplement: Supplementary file 1 [file toxins-18-00001-s001.zip › toxins-4020380-supplementary.pdf]

Supplementary Materials: Influence of Major Environmental Parameters on Patulin Production by *Penicillium expansum* OM1 and its Growth on Apple Puree Agar Media

Haiyi Yu, Sung-Yong Hong, Ji-Yeon Koo, and Ae-Son Om

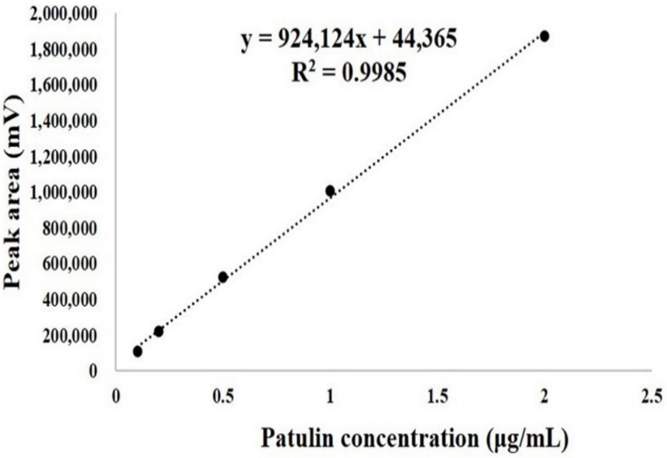

Figure S1. Calibration curve of patulin standard solutions for patulin quantification by HPLC. Five levels of patulin standard solutions were prepared in the range of 0.1, 0.2, 0.5, 1.0, and 2.0 µg/mL. Each solution was injected into HPLC-UVD in triplicate.

A.

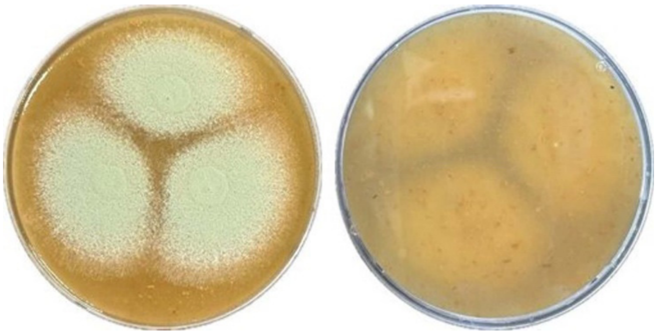

B.

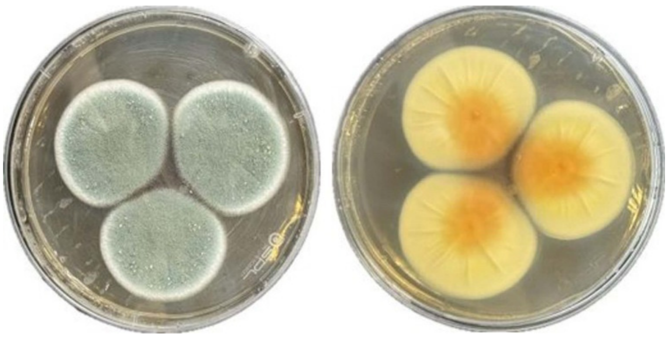

C.

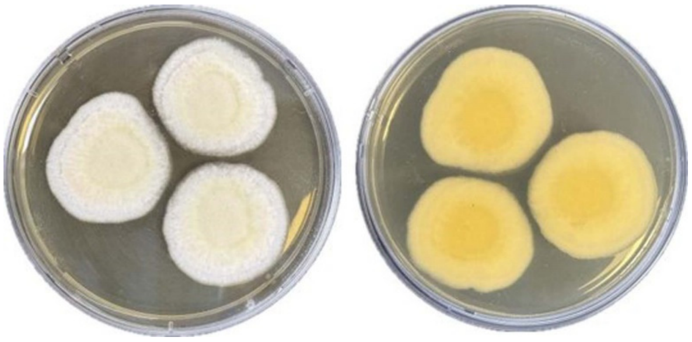

D.

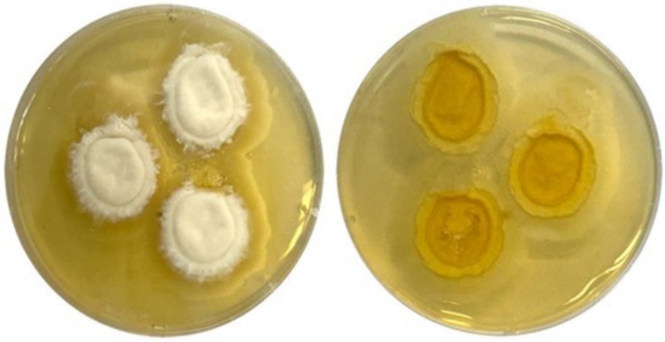

E.

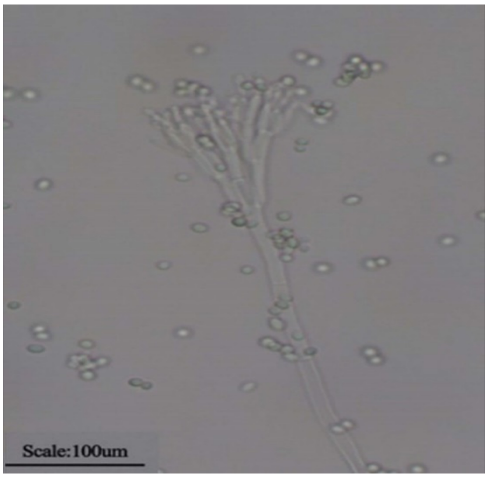

F.

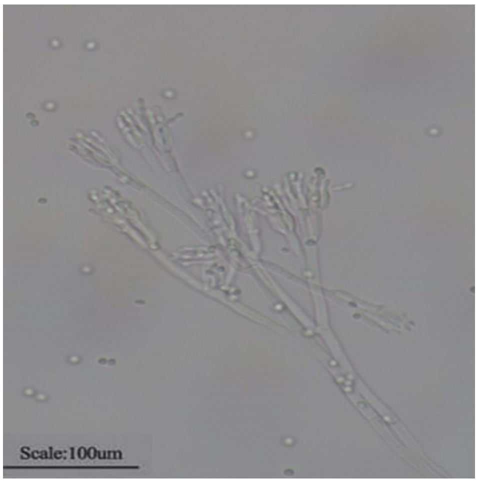

G.

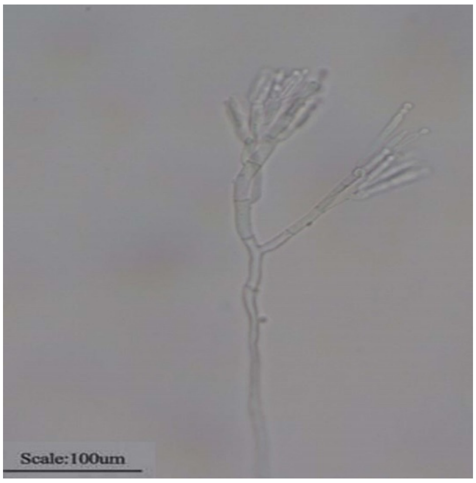

Figure S2. Morphological characteristics of *P. expansum* OM1 on 4 different culture media. (A) Colonies on APAM agar plates, (B) colonies on PDA plates, (C) colonies on MEA plates, (D) colonies on YES agar plates, (E) conidiospores and conidiophore on APAM agar plates (400x), (F) conidiospores and conidiophores on PDA agar plates (400x), and (G) conidiophores on MEA agar plates (400x). Left photographs show top views, while right photographs show bottom views in (A-D). Scale bar, 100 μm.

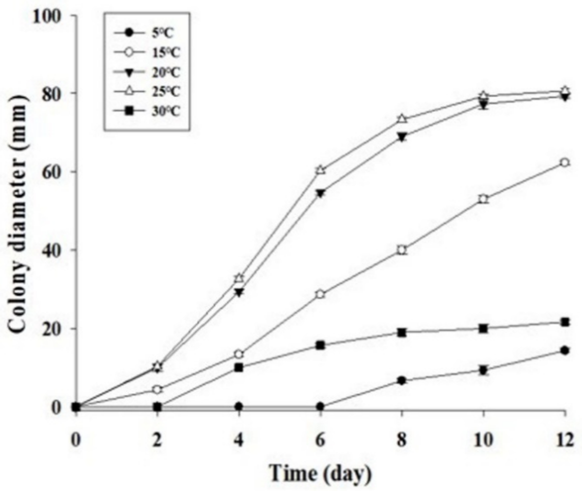

Figure S3. Colony diameters of *P. expansum* OM1 on APAM agar plates under 5 different temperature conditions (5, 15, 20, 25, and 30°C) for 12 days. The radial colony diameter was measured in triplicate. Data are represented as the mean ± standard deviation.

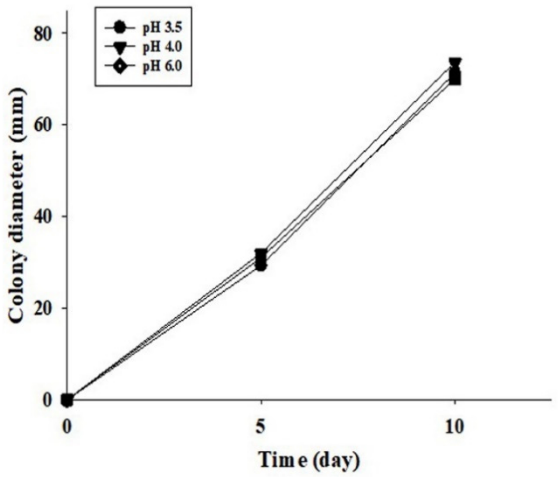

Figure S4. Colony diameters of *P. expansum* OM1 on APAM agar plates under 3 different pH conditions (pH 3.5, 4.0, and 6.0) and 15°C for 10 days. The radial colony diameter was measured in triplicate. Data are represented as the mean ± standard deviation.

A.

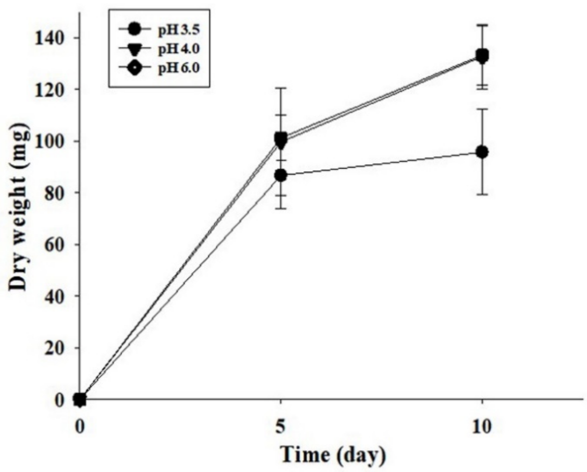

B.

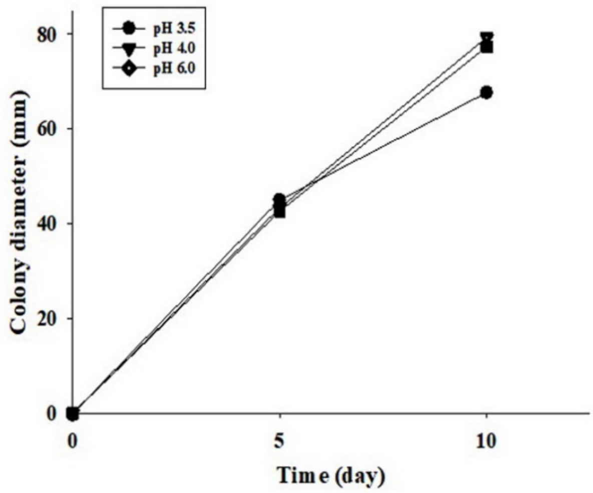

Figure S5. Growth rates of *P. expansum* OM1 on APAM agar plates under 3 different pH conditions (pH 3.5, 4.0, and 6.0) and 25°C for 10 days. (A) Dry weight and (B) colony diameter. The dry weight and colony diameter were measured in triplicate. Data are represented as the mean ± standard deviation.

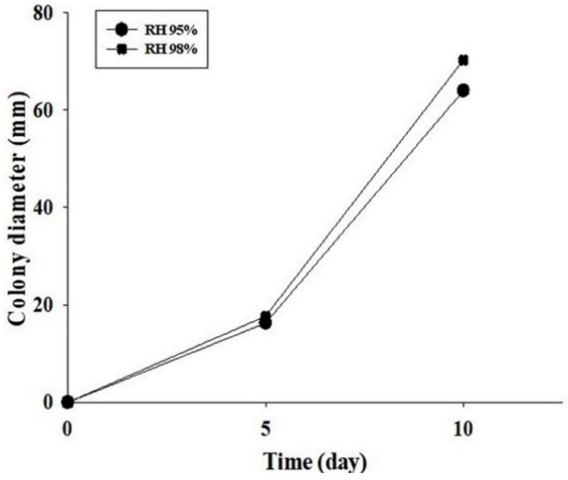

Figure S6. Colony diameters of *P. expansum* OM1 on APAM agar plates (pH 4.0) at 2 different RH (95 and 98%) and 15°C for 10 days. The radial colony diameter was measured in triplicate. Data are represented as the mean ± standard deviation.
